# The cognitive hearing science perspective on perceiving, understanding, and remembering language: The ELU model

**DOI:** 10.3389/fpsyg.2022.967260

**Published:** 2022-09-01

**Authors:** Jerker Rönnberg, Carine Signoret, Josefine Andin, Emil Holmer

**Affiliations:** Linnaeus Centre HEAD, Department of Behavioural Sciences and Learning, Linköping University, Linköping, Sweden

**Keywords:** the ELU model, working memory, semantic long-term memory, episodic long-term memory, adverse listening conditions, age-related hearing loss, dementia

## Abstract

The review gives an introductory description of the successive development of data patterns based on comparisons between hearing-impaired and normal hearing participants’ speech understanding skills, later prompting the formulation of the Ease of Language Understanding (ELU) model. The model builds on the interaction between an input buffer (RAMBPHO, Rapid Automatic Multimodal Binding of PHOnology) and three memory systems: working memory (WM), semantic long-term memory (SLTM), and episodic long-term memory (ELTM). RAMBPHO input may either match or mismatch multimodal SLTM representations. Given a match, lexical access is accomplished rapidly and implicitly within approximately 100–400 ms. Given a mismatch, the prediction is that WM is engaged explicitly to repair the meaning of the input – in interaction with SLTM and ELTM – taking seconds rather than milliseconds. The multimodal and multilevel nature of representations held in WM and LTM are at the center of the review, being integral parts of the prediction and postdiction components of language understanding. Finally, some hypotheses based on a selective use-disuse of memory systems mechanism are described in relation to mild cognitive impairment and dementia. Alternative speech perception and WM models are evaluated, and recent developments and generalisations, ELU model tests, and boundaries are discussed.

## Background

Cognitive hearing science builds on the principle that individual cognitive functions play an important role from very early subcortical auditory processing (Stenfelt and Rönnberg, 2009; Sörqvist et al., 2012) to interactions among memory systems at cortical levels of listening, language understanding, and dialogue (Rudner et al., 2008, 2009; Rönnberg et al., 2021, 2022). Anatomically, several precise downstream corticofugal pyramidal cell axons from neocortical layers 5 and 6 (Usrey and Sherman, 2019) allow for early cognitive impact at subcortical levels, even down to the cochlea (cf. the early filter model, Marsh and Campbell, 2016). This neural organization sets the stage for deep cognitive penetration of the very early sensory and perceptual windows of our experience—a possibility that had not been systematically scrutinized in the audiological and hearing research field before the advent of the Ease of Language Understanding model (ELU, Rönnberg, 2003). Generally, the ELU model (Rönnberg et al., 2008, 2013, 2019) is about rapid abstraction of the meaning of multimodal linguistic input, mediated by working memory (WM) in adverse listening conditions (Mattys et al., 2012).

Our early studies of speech perception and speech understanding focused on speech-reading, or lip-reading (a narrower term not including gesture and body language), adult individuals with normal hearing, impaired hearing, and deafness. The question that we posed was whether persons with hearing impairment—through their increased reliance on visual speech–produced superior, compensatory, visual speech perception/understanding skills. We also investigated the presentation modality, type of materials, type of task (Hygge et al., 1992; even including more ecological tasks Rönnberg et al., 1983), and whether hearing-impairment related variables like the duration of impairment and/or the degree of hearing loss played a role (e.g., Rönnberg et al., 1982, 1983; Lyxell and Rönnberg, 1987; Rönnberg, 1990). The answer was surprising since no compensatory signs were empirically observed.

## The emergence of cognitive hearing science

With further data collections, the data pattern took a radically different turn: First, we tried to examine why some people were such excellent speech-readers (e.g., Rönnberg, 1993; Rönnberg et al., 1999). In a set of case studies of extreme speech-reading skills, we demonstrated that instead of a compensatory effect due to the hearing impairment, it was about cognitive skill in processing and storage of perceived information, measured by the reading span test (RST, Daneman and Carpenter, 1980; Daneman and Merikle, 1996). High RST performance described the cases who in their daily life relied on poorly conveyed auditory speech information, but who still were very competent communicatively: it could be lip-reading only (the case of SJ: Lyxell, 1994), tactile-visually conveyed speech information (the case of GS: Rönnberg, 1993), or a hearing-impaired person with a speech-sign bilingual background (the case of MM: Rönnberg et al., 1999). They all used very different communication strategies, but effectively so. The common denominator of the different case studies reported was that each person was well-equipped cognitively, and that cognitive functions seemed to operate over and above the variables we had studied up to that point. More specifically, it was demonstrated and replicated from the case studies that not only did high WM capacity (WMC) play a significant role in holding information alive, thus presumably mitigating the prediction of upcoming events, but it also represented a cognitive workbench for reconstructing misperceived linguistic units (i.e., postdiction, Rönnberg et al., 2019, 2021, 2022). In the same vein, we found that other related kinds of cognitive functions also contributed to the picture.

It was observed that cognitive functions like lexical access speed (Rönnberg, 1990), executive functions (Andersson and Lidestam, 2005), and inference-making capacity (Lyxell and Rönnberg, 1987) were associated with speech perception and understanding (reviewed in Rönnberg et al., 1998, 2021; Rönnberg, 2003). The data pattern withstood many experimental variations, especially in difficult speech-in-noise conditions (reviewed by Lyxell et al., 1996; Gatehouse et al., 2003, 2006; Akeroyd, 2008; Arlinger et al., 2009; Lunner et al., 2009; Besser et al., 2013), where WMC played the dominating role. Thus, it was (and still is, see e.g., Mishra et al., 2021) hard to escape the general conclusion that poor hearing and/or poorly specified or fragmented speech stimuli depend on individual cognitive processing skills to fill in the gaps of incomplete input to the perceptual and cognitive systems. These findings make up the foundation of Cognitive Hearing Science. For a more complete and historical account of the emergence of the field, see Arlinger et al. (2009).

## Early studies of cross-modal language plasticity

Early neurophysiological evidence spoke to the issue of plasticity of brain tissue and prerequisites for commonalities in central perceptual and cognitive functions. Many studies testified to cross-modal language activations, suggesting for example that visual areas are recruited in pre-lingually deaf cochlear implant users (Giraud et al., 2000, 2001; Zatorre, 2001; Kral and Sharma, 2012). In addition, tactile stimuli in the congenitally deaf tactile aid user activate secondary auditory areas (Levänen, 1998). Also, the duration of deprivation plays a key role in the reorganization of the sensory cortices, such as early sensory deprivation will result in better neural plasticity adaptation (Tillberg et al., 1996; Bernstein et al., 1998; MacSweeney et al., 2001). In normal-hearing listeners, it was suggested that silent lip-reading activates the auditory cortex (Calvert et al., 1997), especially for skilled speech-readers (Ludman et al., 2000). Furthermore, signed and auditory to-be recalled story materials perceived by sign-speech bilinguals (i.e., sign-language interpreters) have been shown to activate temporal areas to a similar extent if a visual component was involved for both modalities (Söderfeldt et al., 1994). However, compared to auditory-only, specific bilateral temporal areas activated by sign-language were involved, specifically the addition of the left area V5, later replicated across imaging techniques (e.g., Söderfeldt et al., 1997; Rudner et al., 2007, but see further under *boundaries*). However, when it came to WM for sign and speech, we still found that there were similarities for left inferior frontal and inferior temporal gyri, which subserve phonological and semantic processing areas (Rönnberg et al., 2004)—areas that also were similarly activated in the early Söderfeldt et al. (1994, 1997) studies. Finally, sign language phonological awareness and word reading ability have also been demonstrated to be associated (Holmer et al., 2016). Again, these data suggest that the brain rapidly transcends the “raw” sensory codes and rapidly abstracts input into modality compatible representations.

In all, there were many early studies that suggested commonalities and plasticity in brain activation independent of language modality and presentation modality. In addition, individual cognitive factors like WMC determined performance on language perception, and the WM system seemed to have modality-independent properties. These neurophysiological data patterns—in combination with the behavioral data—prompted the formulation of a modality-independent ELU model based on individual differences in specific perceptual and cognitive components (Rönnberg, 2003; Rönnberg et al., 2008).

## The ELU model takes shape

In the formulation of the original ELU model (Rönnberg, 2003; Rönnberg et al., 2008), we were quite bold in the sense that the assumption of an occurring mismatch between perceived language input and long-term memory representations of linguistic units was supposed to hold across sensory modalities (auditory, visual, and tactile) as well as language modality (spoken and signed). The “language processor” in the brain was assumed to have a multimodal combinatorial capacity, typically occurring at the “syllabic” or sublexical level across language and presentation modes (Rönnberg, 2003; Stenfelt and Rönnberg, 2009). All cases of mismatch were supposed to trigger an increased dependence on WMC for reconstructive, postdictive purposes.

In some more detail, the ELU system assumes that the perceptual input is conceptualized as an input buffer which Rapidly, Automatically, Multimodally Binds PHOnological information together (RAMBPHO, cf. Baddeley, 2000, 2012; Stenfelt and Rönnberg, 2009). This binding, or integration process, presupposes a rapid “default mode” of abstraction into a multimodal input, where the main task of the system is to implicitly and directly unlock multi-attribute phonological representations in Semantic Long-Term Memory (SLTM), leading to access of lexical meaning (Bernstein et al., 1998; Bavelier and Neville, 2002; Stenfelt and Rönnberg, 2009; Rönnberg et al., 2013, 2019). This process typically occurs during a short time window from 100 to 400 ms, depending on the paradigm, if the chain of events runs smoothly, implicitly, and without effort. In general, this RAMBPHO process is reminiscent of Gibson’s (1966) direct perception approach in that the senses should be considered as interacting perceptual systems, without short-lived intermediary representations.

However, for hearing-impaired participants, or when listening conditions are adverse (e.g., when competing noises or foreign accents are present, or the signal processing in the hearing aid is suboptimal), RAMBPHO-delivered attributes may be fuzzy and too few in numbers to surpass a hypothetical threshold to unlock lexical representations in SLTM (see Rönnberg et al., 2013, for details). The consequence of such a mismatch is that more deliberate, explicit and WM-based storage and processing functions are assumed to be triggered. These WM functions purportedly aim to piece together and infer what was communicated (Lunner et al., 2009; Rönnberg et al., 2013, 2019, 2021). These explicit functions may depend on several inference-making operations within WM but also include several interactions with SLTM and Episodic Long-Term Memory (ELTM), hence taking a relatively longer time than effortless implicit processing. The implicit processes typically operate on a millisecond scale, and the explicit processes may take seconds (Stenfelt and Rönnberg, 2009), and recent evidence suggests that different brain oscillations can dissociate the two (e.g., Gray et al., 2022). There will always be a ratio between the two, which is assumed to vary dynamically from moment to moment due to turn-taking and interlocutor responses in a conversation (Rönnberg et al., 2019).

In general terms, prediction (and postdiction) processes affect the probability that RAMBPHO will match or mismatch with SLTM representations. On a general time scale, RAMBPHO-delivered information always precedes and then affects WM storage and processing operations. If a mismatch occurs, slower postdiction processes in WM feed back to RAMBPHO until comprehension is reached (see more under theoretical implications) or not, for example in cases where the listener is not sufficiently motivated to allocate resources required for further speech processing (Pichora-Fuller et al., 2016). Thus, it is important to acknowledge that RAMBPHO is an obligatory part of an ELU/WM system, feeding linguistic information to the match/mismatch mechanism—which is at the heart of the system. The ELU model describes a communication system which relies on interacting memory systems and mechanisms.

### Experimental evidence

The first experimental manipulations of habitual vs. non-habitual signal processing in hearing aids successfully tested the cognitive consequence of the mismatch notion. We developed several different kinds of methods to trigger a mismatch. One example, and probably the most evident demonstration, was that of the studies by Rudner et al. (2008, 2009). For example, experimental acclimatization to a non-habitual kind of aggressive hearing-aid signal processing for 9 weeks (i.e., FAST or SLOW wide dynamic range compression) and then subsequent testing in a previously non-acclimatized mode of signal processing (i.e., SLOW-FAST or FAST-SLOW), produced strong reliance on WM in those two mismatching conditions, compared to the matching FAST-FAST and SLOW-SLOW conditions. As a matter of fact, the effect of just shifting from the regular hearing-aid settings to new ones produced higher reliance on WMC (for replications and relevant supporting studies/reviews, see Souza and Sirow, 2014; Souza and Arehart, 2015; Rönnberg et al., 2019; Souza et al., 2019). These findings also expose the interplay between WM and SLTM in the development of representations, as introduced to the ELU framework in recent years (Holmer et al., 2016; Holmer and Rudner, 2020; Rönnberg et al., 2022). In response to a novel input signal, such as that produced by new settings in a hearing aid or learning a new word, the language system is likely to treat the input as something unfamiliar, i.e., a mismatch condition, which WM resources are used to solve. However, each time a mismatch condition is resolved, this has the potential of producing an adjustment to the exemplars associated with the representational space in SLTM.

A second example is the investigation of competing effects of different kinds of maskers. Many studies agree with our view that so-called energetic maskers (Brungart, 2001) produce distraction but not to the same extent as informational maskers engaging SLTM (e.g., Rönnberg et al., 2010; Mattys et al., 2012; Sörqvist and Rönnberg, 2012; Kilman et al., 2014), and that distraction is more pronounced if the masker was in the participants’ native language (Kilman et al., 2014; Ng et al., 2015). It should be noted that the original data of WM dependence (using “speech-like” maskers) had already been observed and discussed (Lunner, 2003; Lunner and Sundewall-Thorén, 2007; see a review by Rönnberg et al., 2010). Again, in retrospect, the effects of informational or speech-like maskers were related to partial activation of SLTM, e.g., phonologically similar neighbors (Luce and Pisoni, 1998) or possibly, with ELTM repetitions of SLTM contents.

Thirdly, as noted above, WMC is also an important predictor of performance in ELTM in such circumstances of initial speech-in-speech maskers (e.g., four-talker babble, 4T, Sörqvist and Rönnberg, 2012; Ng and Rönnberg, 2020). Notably, the robustness of the high dependence on WM was not influenced by the duration of hearing-aid use, at least up to 10 years of hearing-aid use for four-talker (4T) maskers (Ng and Rönnberg, 2020). This raises the question of whether some kinds of conversational environments are too dynamic to allow for a lessened dependence on WM. That is, highly dynamic input—or input with poorly defined phonological information—might stress the boundaries of how well the system can adjust its representational space (Han et al., 2019). The 4T-masker results hold irrespective of signal processing in the hearing aid. However, contextual support (Rönnberg et al., 2016) or plausibility/predictability of sentences may override the need for WM resources (Moradi et al., 2013; Amichetti et al., 2016), which in turn makes the signal-context interactions determine the potential need for postdictive processing. The overall idea is that the brain should not invoke WM resources unnecessarily, e.g., when context drives a more rapid and implicit route to comprehension. In that sense, the brain is “lazy” and economical in spending effort and processing energy, using a principle of least effort (cf. Ayasse et al., 2021; Silvestrini et al., 2022).

A final study points to constraints when bimodally combining CI-listening in one ear with listening with a hearing aid in the other (Hua et al., 2017). These two types of signals reaching the brain will not necessarily be RAMBPHO compatible. From an ELU perspective, an electric and a physical-neurostimulation might be harder to convert into some more abstract representation than naturally occurring multimodal sensory stimuli. Analogous to habitual vs. non-habitual signal processing in the hearing aid, this combination of inherently different signals to the brain does not seem to combine easily. One indication is that RST was the most sensitive predictor variable for bimodal sentence materials compared to unimodal listening conditions, where the trail-making test (primarily measuring cognitive speed) was more critical to unimodal conditions and single word identification (Hua et al., 2017). But, it should also be noted that the bimodal condition facilitated speech-in-noise performance, although the cost in terms of WM engagement and effort may create a balancing act that the individual and clinician must decide from the individual WMC data.

Related to these difficult (or mismatching) speech processing tasks is a couple of recent studies corroborating the ELU hypothesis about the engagement of WM in challenging listening conditions. Mishra et al. (2021) found that WMC accounted for large portions of variance (up to 80%) of word recognition in speech noise of spondees and phonetically balanced word lists presented at dB SNR 0, –10, and –20, whereas in quiet the pure tone average explained 78% of the word recognition scores and not the RST scores (see also Kurthen et al., 2020). However, there is also evidence for specificity in correlations between the demands of the WM task and the complexity of the criterion task (Heinrich et al., 2015; see Rönnberg et al., 2021 for a discussion). Thus, the actual principle of mismatching is replicated in Mishra et al. (2021) with the concomitant demand on WM resources, but further task analyses of both tests of WMC and the type of outcome remain to be carried out (Heinrich et al., 2015). Nevertheless, see our first attempts involving interactions among memory systems (Rönnberg et al., 2011, 2014, 2021).

### Linguistic abstraction and WM capacity

In gating tasks (Grosjean, 1980), the participants are required to identify a consonant/vowel or a final word in a sentence, based on the presentation of successive bits of initial phonetic information of the speech token (Moradi et al., 2013, 2014a,2014b). These studies have demonstrated that early and successful linguistic identification requires WMC. WM is involved in the rapid identification of e.g., a consonant when the semantic context is lacking. Around 90 msec is necessary to identify speech tokens in the auditory gating paradigm (Moradi et al., 2014b). Audiovisual presentation reduces this identification time to 40–50 msec, as does identification of final words in highly predictable sentences (Moradi et al., 2013). This time reduction presumably implies very rapid neuronal communication between different brain regions that, in turn, activate different memory systems. However, hearing loss (even when compensated with hearing aids), age, and noisy signals, all slow down the identification process (Moradi et al., 2013, 2014a).

A further example of rapid abstraction is a kind of priming paradigm which has demonstrated the so-called “pop-out” effect (see a general discussion in Davis et al., 2005), where presenting the written version of a sentence on a computer screen creates an enhanced perceived clarity and understanding of spoken, noise-vocoded, sentences that are otherwise incomprehensible due to the vocoding. In the studies by Signoret et al. (2018), Signoret and Rudner (2019), the written version of the sentence was presented word by word (200 msec before the vocoded version) until a sentence was complete, and required the participant to rate the perceptual clarity of the vocoded sentence. Perceptual clarity is enhanced for semantically coherent and phonologically primed sentences, but when compatibility is low, WM processes entered into play. For example, WM was invoked in conditions with non-matching primes, substantial vocoding, and low semantic coherence (Signoret and Rudner, 2019).

In a recent study using magnetoencephalography (MEG, Signoret et al., 2020), participants were to decide whether the final word in a sentence was expected or not. The participants had studied the sentences before the actual experiment, so expectations on the final word were strong. Different kinds of deviants were used as final words (in background noise): either the final word was semantically different but rhymed with the expected word, or was semantically related but did not rhyme, or was different in both aspects of similarity. Notably, WMC negatively correlated with the number of false alarms to meaning deviants that rhymed, such that participants with high WMC were less lured into accepting a deviant via the RAMBPHO to the SLTM matching process. Further, participants with higher WMC had processed meaning deviants more easily (smaller N400 effect) compared to participants with lower WM capacity. Participants with high WMC, also processed the semantic mismatching more easily (smaller N400 effects) and showed better performance at the behavioral level. WM therefore seems part and parcel of the prediction mechanism.

In sum, the hypothesis of RAMBPHO-like multimodal representations that together with high WMC activate SLTM, receive support from the above examples.

### Theoretical implications

Theoretically and neurophysiologically, the RAMBPHO-SLTM interaction necessarily utilizes very rapid subcortical and cortical connections that allow for the implicit initial matching process. At this stage, the *prediction* aspect of the ELU model allow for matching processes most likely affecting attention to certain aspects of the input signal, which naturally varies in form, content, and modality (Samuelsson and Rönnberg, 1993; Sörqvist et al., 2012; Sörqvist and Rönnberg, 2012). Because of the correlations with WMC cited above, we can assume that RAMBPHO rapidly “constructs” a multimodal channel to WM that matches multi-attribute or multimodal representations in SLTM. Thus, a high WMC may facilitate early attention fine-tuning of auditory processing but may also reflect a highly synchronized brain network (Fell and Axmacher, 2011). The conclusion about some kind of early fine-tuning is reinforced by the finding that WM processes are positively interconnected with the effects of practice on auditory music skills (Kraus and Chandrasekaran, 2010) and their corresponding neural brain stem signatures (Kraus et al., 2012).

Thus, we argue that the brain always has, as its primary aim, to abstract meaning, i.e., a cognitive hearing, sense-making, organism. This aim holds regardless of whether the stimuli are represented by sublexical or lexical items, or grammatical constraints in a sentence (Ayasse and Wingfield, 2020). And, if there is a mismatch, there is the advantage of already existing multimodal representations maintained in WM—given a sufficiently capacious system—which can be deconstructed and/or reconstructed among brain networks that belong to SLTM and ELTM (i.e., postdiction).

Furthermore, since we initially have a presumed representation which is rapidly constructed but which also can be deconstructed by cues, and then reconstructed again, perhaps several times, it necessarily takes longer to execute explicit interactions among brain networks of the brain that belong to the WM, SLTM, and ELTM systems. The explicit postdiction process may take seconds, while the prediction process is on the msec scale. The neural mechanism diverges between them: while the postdiction process is proposed to relate to theta-activity, the prediction process is proposed to relate to alpha-activity (Gray et al., 2022). Typically, the postdiction process becomes more explicit and slower the neural process (i.e., probably related to enhanced theta activity), whereas the prediction processes are more implicit and probably related to faster neural activity, such as decreased alpha activity (Gray et al., 2022) or beta activity (Signoret et al., 2013). Although predictions are often primed implicitly in everyday conversation in their effect on RAMBPHO, contextual cues can be explicitly held in WM prior to the experimental materials (Zekveld et al., 2013), in a similar vein to when postdiction feeds back into RAMBPHO. This priming mechanism likely aims to pre-activate specific knowledge at different levels of processing depending on the environmental context and on individual abilities and skills. Generally speaking, the ELU model assumes an overarching prediction-postdiction system, both dependent on WMC, albeit in different ways (Rönnberg et al., 2019, 2022).

### Output from the ELU system

The output of the ELU system is lexical access, the grasp of what was communicated, and what consequently may be recalled from ELTM. Further, the output might involve a change to SLTM, either during development (Holmer et al., 2016) or when adjusting to novel listening conditions (e.g., Ng and Rönnberg, 2020). We have also shown that WMC contributes to ELTM performance in terms of sentence recognition (e.g., Sörqvist and Rönnberg, 2012; Zekveld et al., 2013). In Sörqvist and Rönnberg (2012), it was demonstrated that performance on the Size Comparison (*SIC*) span WM test predicted higher immediate and delayed recall of fictitious stories masked by a person reading from another story at encoding—*SIC* span made significantly better predictions in hierarchical regression analyses the RST. This presumably comes from the fact that apart from processing and storage, as in the RST, *SIC* span involves an additional inhibition component. For example, the task could be to compare four-footed animals in a list of comparisons and an additional to-be-remembered word for each comparison: Is a zebra larger than a mouse? (the Comparison) + the word (lion). Each list of comparisons and words belong to the same semantic category, which could cause confusion between comparison words and to-be-remembered words at the recall of the list. Obviously, an inhibition factor comes into play, and mediates better recall.

Although not a prime purpose of that study (Sörqvist and Rönnberg, 2012), the clinical implications are important as well. As it happened in this study, WMC was important for both immediate performance and for ELTM. The ELTM aspect is crucial from a listening perspective. If the actual signal processing in the hearing aid or the adversity of the listening situation drains too much processing capacity in the “here and now” situation, then less is left over for storage. This implies that conditions for change might also be circumscribed in noisy conditions, perhaps because little WM resources are left for successful encoding into change and development of SLTM. To compare with a long discussion about task demands on storage and processing, see Lunner et al. (2009) and Rönnberg et al. (2021).

In other words, in the dialogue between a hearing-impaired person and an interlocutor, the hearing-impaired person must allocate explicit attention to mismatches when they occur to be able to extract meaning, and perhaps learn something new, from the conversation. This is not required of the normal hearing person to the same extent. This is exactly the reason why a measure of what is left in ELTM after smaller amounts of storage resources remain in WM (e.g., tested a day or two after the conversation) would clinically be a very important ecological aspect of what it means to approach ease of listening and understanding for a hearing-impaired person. Without going into detail, a test that tapped into storage, semantic processing functions, and inhibition at the same time, would seem to be a suitable candidate based on the data we have collected (e.g., Sörqvist and Rönnberg, 2012; Stenbäck et al., 2015). Furthermore, what you remember from a conversation has obvious personal and social consequences, and such consequences might sometimes lead to developing depression (Keidser et al., 2015).

In further support of the inhibition component, recent studies by Stenbäck et al. (2015, 2021) verify that especially WMC (measured with the RST) but also the Swedish Hayling test (Stenbäck et al., 2015), which measures inhibition, were significant predictors of performance in speech-in-noise (SPIN, Hällgren et al., 2006) and Hagerman matrix sentences (Hagerman, 1982). The Hayling test builds on the ability to inhibit sentence completion of the last semantically correct word instead of providing a semantically incorrect but grammatically correct word. Thus, WMC and executive functions are part and parcel of listening, understanding, and recalling in adverse conditions.

In general, inhibition and turn-taking in real dyads or conversation/discussion groups put large demands on the timing of turns. If you are, e.g., interrupting too many times, it might just be the case that you do not have sufficient WMC to follow the line of thought in the conversation. To do that, you need to keep in mind what was just said and process it, at the same time as you are planning to latch on with your own turn, and what you are going to respond to. Therefore, taking another person’s perspective in a dialogue demands WMC functions of maintenance, timing and dual storage and phonological/semantic processing. However, taking the perspective of someone else also involves the cognitive function of theory-of-mind (ToM), which is about decoding and understanding other people’s intentions and feelings, and not necessarily just grasping what was actually said in the conversation (Hagoort and Levinson, 2014). That is, the intended meaning might sometimes not be coded in the meaning of the exact wording of a sentence, and therefore, ease of language understanding is about multilevel understanding in dialogue.

## Recent findings

Füllgrabe et al. (2014) and Füllgrabe and Rosen (2016) claimed that WMC only accounted for significant amounts of variance in elderly hearing-impaired participants’ SPIN performance, whereas for younger normal-hearing participants, only a small percent of the variance was accounted for by WMC. Nevertheless, Vermeire et al. (2019) clearly showed that for elderly normal-hearing participants, RST was a significant predictor of SPIN performance as well. Indeed, Gordon-Salant and Cole (2016) showed the same results to hold across age groups, with RST as part of the most prominent predictors. In the same vein, with large samples, Marsja et al. (2022) used the n200 database (Rönnberg et al., 2016) to study potential differences in cognitive involvement due to hearing loss. Marsja et al. (2022) used a multi-group structural equation model (SEM) approach where the purpose was to assess whether the contribution of a “Cognition” latent variable (based on RST, a visuospatial WM test and a semantic WM word-pair test, and Raven’s matrices) was equally related to a SPIN criterion (Hagerman matrix sentences, Hagerman, 1982) for hearing-impaired hearing-aid users compared to normal-hearing participants. The results, based on 200 participants per group, show that the Cognition variable accounted for identical beta weights (-0.32) in both groups of equal average age (60 years), when the groups were compared on an outcome latent construct based on Hagerman matrix sentences. Thus, the cognitive contribution to SPIN perception is not specific to elderly hearing-impaired participants. The statistical models were partialed out for age and hearing loss, and significant on all relevant model fit parameters. This is generally supportive of the initial claims of the ELU model, viz. that there is a communality in cognitive abstraction and cognitive prediction across adult groups with different hearing status (Rönnberg, 2003).

### The devil is in the details

It is important to note that the interaction between the fine details of task demands may make a large difference in terms of predictability of outcome in a SPIN task. For example, in the original RST (Daneman and Carpenter, 1980), participants always recalled the final words in each sentence set—which obviously invites a strategic component compared to the version we use (Rönnberg et al., 2016), where participants are post-cued to recalling *either* the first *or* the last word of the sentences to be verified. In the latter case where the strategic component is reduced, the “raw” WMC is more likely to be revealed. Our research builds on this latter task version and that “detail” may be a clue as to why some researchers get higher involvement of WM than in some other studies (e.g., Ng et al., 2013; Souza et al., 2019; Ng and Rönnberg, 2020). Other aspects relate to contextual support either at the prediction stage or in the sentence materials themselves; high contextual support renders lower correlations with WMC, and vice versa (Moradi et al., 2013; Rönnberg et al., 2016). Dependence also varies with age, hearing status, and a host of other factors related to hearing aid signal processing and habitual processing demands, and not least the interplay amongst the speed, phonology, and WM factors depends on the level of adversity of the listening situation (Homman et al., submitted).

### Structural equation modeling

In a recent study by Homman et al. (submitted) on the hearing-impaired participants in the n200 study (Rönnberg et al., 2016), we used Structural Equation Modeling (SEM) based on the original cognitive parameters in the ELU model: speed, phonology, and WM (Rönnberg, 2003). Thus, one latent speed parameter (i.e., physical matching and lexical access speed), one latent phonological parameter (auditory and audiovisual gating conditions and rhyme tests, i.e., measures of RAMBPHO), and one latent WMC parameter (i.e., based on the RST, visuospatial WM, and semantic word-pairs), were included, while age and hearing loss were partialed out. The results show that phonology always contributed to the performance in the different Hagerman conditions (irrespective of noise type, performance level, and type of signal processing). Speed did not directly predict the Hagerman outcome, but speed always predicted WM, and the WM to Hagerman path was significant only in the more difficult listening conditions involving 4T maskers. Thus, the new and interesting result of this mediation analysis was that speed contributed *via WMC* to Hagerman in the difficult conditions, i.e., where higher degrees of mismatch can be assumed. The general interpretation is that when being exposed to adverse listening conditions, it is important that WM is capacious because it takes more time to reconstruct what was perceived, i.e., when a more laborious explicit mode of processing is needed. An alternative interpretation would be that the adversity of the listening situation primarily strikes at RAMBPHO. Nevertheless, optimizing speed in WM operations becomes critical in both cases. This result agrees with previously reported results in Rönnberg et al. (2016), where WM was more strongly related to Hagerman matrix sentences than to HINT sentences, which are contextually driven everyday sentences. By virtue of the semantic coherence in HINT sentences, the prediction mechanism is improved, hence lessening the demand on WM resources for postdiction (cf. also Moradi et al., 2014b).

In a similar SEM approach, Janse and Andringa (2021) modeled word recognition performance in degraded low-pass filtered conditions. They used cognitive speed, vocabulary, hearing acuity, and WM as latent constructs. In their model, WM was the strongest latent construct relating to word recognition in noise, replicating our research. The RST was the test that loaded the highest on the WM factor, compared to digit span and non-word recall (cf. Rönnberg et al., 2016). In our current model (Rönnberg et al., 2021, 2022) we only used WM tests that emphasize storage and processing in dual task formats. We noted that speed of access from SLTM was our mediating factor. However, their mediation model of vocabulary via WM to word recognition (Janse and Andringa, 2021) is not directly comparable to ours, as we did not use vocabulary, but interesting indeed. The communality is that WM predictive capacity is only predictive of SPIN performance, via some back-up parameter such as SLTM speed or SLTM vocabulary. It is obvious that these mechanisms support WM when more complex interactions between WM, SLTM, and ELTM are required for postdiction purposes.

## Comparison with other models

### Perception and understanding: multimodal and multilevel aspects

Speech perception models are less comprehensive than the ELU model, but in some cases more specific. For example, in the Neighborhood activation model (NAM model, Luce and Pisoni, 1998), lexical access is clearly dependent on how input stimuli matches/mismatches with the lexicon due to phonological similarity and semantic parameters like word frequency. In the initial word cohort model (e.g., Marslen-Wilson, 1987), the initial information that enters the ear is assumed to activate a set of competitors in a cohort of possible candidates, a functional parallelism in the activation of the lexicon. As information successively enters the auditory system, activation and selection of candidates proceeds until only one lexical candidate remains. There are many manipulations of the selection process e.g., by priming, word length, or word endings that in different ways manipulate the word recognition point, which often occurs before the whole word has been perceived.

Furthermore, the probability of lexical access is not an all or none process (as described in Rönnberg et al., 2013); it depends on the RAMBPHO input and the kinds of representations it meets in LTM. And, at some hypothetical threshold of matching attributes lexical access is triggered. The types of error responses we obtain may well be captured by the NAM (Luce and Pisoni, 1998), but in addition we have also made clear that the prediction- postdiction cycle may prime or direct the individual ELU system to other aspects of representation in LTM that then helps the system to surpass the threshold and retrieve the correct lexical candidate. A good example of such priming by sentence context can be found in a MEG study by Signoret et al. (2020), where phonological and semantic error responses were in focus.

Also related to RAMBPHO, we focused on a special form of priming. We dubbed the hypothesis “perceptual doping” (Lidestam et al., 2014; Moradi et al., 2019). In brief, the priming effects of exposure to two initial conditions (auditory only, or audio-visually presented materials) on later auditory perception of consonants, vowels, and sentence materials generally demonstrated a multimodal facilitation (“doping”) effect. The interpretation is that there is a recalibration/remapping of the initial audiovisual presentation mode affecting the SLTM representation of phonological and lexical attributes. With the advantage of hindsight, a discussion of the data based on RAMBPHO may also have been possible.

Related to our mismatch concept, earlier basic auditory perception studies outlined the basic properties of the mismatch negativity (MMN) effect measured with EEG (Näätänen, 1995; Näätänen and Escera, 2000). In our research, we have emphasized the *consequence* of mismatch in terms WM involvement (Rönnberg, 2003). Relevant to the current paper is that the mismatch notion has also been applied to grammatical levels of language processing (Federmeier, 2007), as well as phonological/semantic processing (Signoret et al., 2020). The ELU model is here proposed to be about levels of linguistic mismatch, from RAMBPHO and lexical access, via grammar to semantic coherence. Therefore, the fact that the functional role of the frontal cortex in pre-attentive auditory change detection has been shown for grammatical deviations is of high importance (Hanna et al., 2014). The mismatch negativity function automatically detects grammatical anomalies around 200 msec, after the grammatical violation point (subject–verb agreement violations or word category violations, Hasting et al., 2007; cf. Signoret et al., 2020 for different violation types). Tse et al. (2013) and Hanna et al. (2014) have both demonstrated and discussed the very early, pre-attentive and automatic Broca/inferior frontal signals of mismatch negativity for grammatical violations (around 200 msec), which could be an inspiration to our new, more elaborated ELU proposal, of co-occurring mismatch signals possible at different linguistic levels (see below).

In our current view, the multimodal phonological level is crucial to SPIN performance, but if implicit processing occurs at higher levels of language like syntax, keeping auditory characteristics under control (Hasting et al., 2007), it makes our claim about the necessity of rapid WM interactions with SLTM and ELTM even more important. Otherwise, these extra steps would probably prolong the extra time for reconstruction and postdiction. This specification of the ELU model is that not only is WM involved in rapid RAMBPHO-delivered multimodal abstraction, but it is also involved in multilevel language interactions, given that there is some central mismatch time widow for several levels of language, and which can be processed in parallel (cf. Marslen-Wilson, 1987). *We submit that the prediction-RAMBPHO-SLTM-postdiction interaction demands a “moving time window” within the confines of WMC, the contents of which are rapidly abstracted at multimodal and multilevel aspects of input. In a more generalized form, it may be stated that: for any given aspect of RAMBPHO-delivered linguistic information, the cognitive consequence of a mismatch with SLTM representations, is bound to initiate WM-based postdiction.*

Furthermore, the cognitive consequence of a central multimodal/multilevel mismatch mechanism has not been fully realized in the ELU model, but comparisons with Central Auditory Processing Disorder (CAPD) research could inspire (Gates et al., 1996; Gates, 2012). However, several such multimodal and multilevel interactions will need to exploit all the storage and processing capacities of WM. Therefore, the postdiction processes will necessarily take more time than implicit predictions. But if there are rapid multilevel mismatch functional capacities of the brain, it will allow for an advanced analysis-by-synthesis kind of model, demanding such on-line revisions of what is misperceived (cf. Hickok and Poeppel, 2007). It also demands parallel processing not just at the word level. For example, in the Moradi et al. (2014b) study, high predictability sentences were completed with only minimal initial phonemic information of the final word (40 msec).

Thus, we still assume that relatively context-free perception is dependent on RAMBPHO-based lexical retrieval. But, context-bound, grammatically incorrect sentences can also induce mismatch at some violation point in the sentence. This implies that the functional parallelism (cf. Marslen-Wilson, 1987) is not only realized through multimodal streams of information, as in the ELU, but also in parallel streams at different levels of language that act in concert to optimize implicit understanding of the discourse. This may at later stages demand cognitive functions to keep track and focus on the “winning stream” of information processing (cf. Moradi et al., 2013). Again, presumably the brain is optimizing speed of mental operations in WM even in mismatch situations. However, to our knowledge, the mismatch studies have not emphasized the communicative feedback, which the ELU model denotes as postdiction, which in turn is assumed to feed back into predictive RAMBPHO processing. This postdiction feedback may not only alter predictions but also induce SLTM changes.

Thus, even when well-organized and linguistically interactive and smooth processing is taking place, mismatch at some linguistic levels will demand reconstruction and postdiction, typically taking more time. Parallel levels of processing (without any mismatch) may on the other hand synergistically integrate input and reduce processing time (Moradi et al., 2014a,b; Signoret et al., 2020).

### Working memory

In comparison with other working memory models, the ELU model is very much inspired by two working memory traditions, the Baddeley and Hitch (1974; Baddeley, 2012) tradition and its many developments (e.g., the episodic buffer Baddeley, 2000, cf. RAMBPHO), as well as the tradition following a more general resource model tradition, with less structural assumptions on dedicated loops and modular functions (cf. our use of the RST, Daneman and Carpenter, 1980; Just and Carpenter, 1992; Daneman and Merikle, 1996; Barrouillet and Camos, 2020).

More specific capacity models sometimes have taken the form of activation of LTM relevant information, not seldom related to expertise (Ericsson and Kintsch, 1995; Cowan, 2005; Jones et al., 2007). The activation capacity of several representations in LTM thus becomes a measure of expertise, or WMC. In the ELU model, there are two roads to LTM in principle, one implicit and one explicit. This conceptualization and difference to the above models is dependent on the assumption that the ELU model is primarily conceived for communication purposes, where mismatch disturbs the flow of rapid phonologically mediated lexical access, but where WM must engage SLTM and ELTM to optimize explicit postdictions, as well as predictions. Seen from this horizon, the ELU model captures what we believe to be a human propensity, viz. the system is “lazy” or economical (cf. Richter, 2013); it does not spend explicit resources unless sub-threshold levels of language input cause mismatch (especially the phonologically mediated lexical access function).

There have been several recent attempts at refining the component concepts of the ELU-model. The ELU-model has generated several important scientific hypotheses and ways of investigating and testing them.

### Model refinements

Edwards (2016) suggests that just before RAMBPHO processing occurs, a process is needed that accomplishes early perceptual segregation of the auditory object from the background, so called Auditory Scene Analysis (Dolležal et al., 2014). His discussion is based on the Rönnberg et al. (2008) version of the ELU model, where RAMBPHO processing focuses on how different streams of sensory information are integrated and bound into a phonological representation (see also Stenfelt and Rönnberg, 2009). Nevertheless, in Rönnberg et al. (2019, 2022), it is made more explicit that the system may feedback via postdiction processes, which may prime the prediction process (Sörqvist and Rönnberg, 2012), including fine-tuning of attention (Holmer and Rudner, 2020; Andin et al., 2021) and selection processes to specific features of the input (Rönnberg et al., 2013). This seems to be rather close to stream segregation, but the theoretical languages differ. By inference, postdiction may then calibrate the selection of the auditory object, comparable to “perceptual doping” (Moradi et al., 2019).

The second aspect is that RAMBPHO is assumed to be primarily dedicated to phonologically relevant information, embedded in lexical and semantic representations in SLTM. Lexical access and semantic meaning of sentences are tightly tied to the mismatch mechanism—and by default—finding of a linguistic object. Thus, that aspect of Edwards (2016) proposal does not necessarily demand model change (Rönnberg et al., 2019).

In the D-ELU model (Holmer et al., 2016), the development of language representations in SLTM is in focus. The original ELU model focused on the system’s input side and the WM-LTM interactions, but the development of appropriate SLTM representations has hitherto received less interest. Nevertheless, it has been demonstrated that vocabulary is very important to speech perception in noise (Kennedy-Higgins et al., 2020), either via WMC (cf. Janse and Andringa, 2021), for hearing-impaired listeners (Signoret and Rudner, 2019), or in how language is represented in bilinguals (Kilman et al., 2014; Bsharat-Maalouf and Karawani, 2022). According to the D-ELU model, existing lexical representations in SLTM shapes further lexical growth, i.e., novel representations build upon existing representations (cf. Jones et al., 2021). Novel words that are rich in lexical attributes are more likely to be successfully encoded into SLTM, and thus learning rates are predicted to be steeper. Further, learning for persons with hearing loss is predicted to be worse than for controls when the perceptual platform at the learning stage is too dynamic (Ng and Rönnberg, 2020).

In the study by Kilman et al. (2014), and of relevance for how representations develop, we found in Swedish native speakers who also knew English, that the most interfering speech in noise condition was when the speech masker was in the same (Swedish) native language as the target. The Swedish babble was interfering more than the English babble in stationary noise, and in fluctuating noise. The interference from language maskers replicates previous work (Van Engen and Bradlow, 2007; Calandruccio et al., 2010).

A recent study by Bsharat-Maalouf and Karawani (2022) examined the speech perception of 60 Arabic-Hebrew bilinguals and a control group of native Hebrew speakers during degraded (speech in noise, vocoded speech) and quiet listening conditions. There was a clear interaction in the data such that performance in the bilinguals was on a par with the native Hebrew speakers in quiet conditions, whereas performance in the babble noise conditions (same language of the noise and targets) was substantially lower. Explaining these and other effects, in terms of proficiency (Kilman et al., 2014) of second language, age of acquisition, propensity to learn in vocoding conditions (Bsharat-Maalouf and Karawani, 2022), and what kinds of SLTM representations mediate these findings is of importance for the bilingual and developmental aspects of the ELU model. Future publications will tell.

## Aging, cognitive impairment, and dementia

The ELU emphasis on a meaning-related focus of the brain’s perceptual-cognitive system is assumed to prioritize multi-attribute representation and multilevel mismatch processing. In other words, both children and adults are primarily tuned in to understanding language and intended communication but can of course be instructed to learn or memorize what has been communicated. In terms of a use/disuse principle (Rönnberg et al., 2011, 2021), WM is on top, always dealing with both pre- and postdiction processes on-line; the next memory system is SLTM due to the natural semantic bias in interpretation of conversation and discourse, and ELTM will be relatively less used for two reasons: (1) a non-prioritized bias in communication, and (2) denied encoding and retrieval due to hearing loss or other adverse conditions. Thus, disuse can be a key to why WMC is relatively spared when it comes to cognitive decline studies (Rönnberg et al., 2011, 2014), whereas semantic and especially ELTM decline becomes a marker of mild cognitive impairment, which might develop into dementia.

The disuse notion of memory systems is mainly supported by two major studies by our team: (1) In Rönnberg et al. (2011), based on the Betula prospective cohort study (Nilsson et al., 1997), we found that hearing loss did not selectively affect different ELTM encoding tasks in different sensory modalities (i.e., motorically, by text and simultaneous auditory presentation, and auditory only compensated with hearing aids). If anything, the hearing loss–ELTM encoding task correlations were higher with the motorically encoded task. This may seem counterintuitive, unless one assumes multimodal representations and that the multimodal memory system level is negatively affected, not memory via a specific encoding modality. (2) In addition, long-term memory, especially ELTM was affected by hearing loss, but not by visual impairment. The fact that the cognitive aging and dementia-related literature suggests that ELTM is the most sensitive predictor variable among memory systems to mild cognitive impairment and dementia (Bäckman et al., 2001; Fortunato et al., 2016; Younan et al., 2020) makes our case strong. Combining (1) and (2), we may infer that hearing loss is an important risk factor for accelerated dementia progression (Livingston et al., 2017).

Common cause accounts (e.g., Baltes and Lindenberger, 1997; Humes, 2013; Powell et al., 2021) may predict that hearing loss affects several encoding modalities, but they do not predict selectivity of memory systems. In Rönnberg et al. (2014)—building on 138098 participants from the UK Biobank resource—we observed that ELTM was more affected by hearing loss than WM, thus replicating the data from Rönnberg et al. (2011). In terms of encoding modality, the Rönnberg et al. (2014) study employed visuospatial tests only. Still, we obtain a negative effect of hearing loss on ELTM and not on WM. This further supports and replicates a memory systems account of relative use/disuse as a potentially viable ELU explanation of the data pattern.

In addition, the potential risk of cognitive decline due to hearing loss cannot be explained by the information degradation hypotheses (Pichora-Fuller, 2003; McCoy et al., 2005), nor by attention costs for the hearing-impaired person (Sarampalis et al., 2009; Tun et al., 2009; Heinrich and Schneider, 2011). This could have been the case had the auditory encoding condition been negatively affected by hearing loss, even if the participants wore hearing-aids at testing (Rönnberg et al., 2011). A further important aspect of the 2011 data is that testing the same models, replacing hearing loss with estimated visual impairment (legibility of font size, on a scale from 6 to 24, Rönnberg et al., 2011; wearing eye glasses or not, or having a diagnosis, Rönnberg et al., 2014), did not replicate the memory system selectivity of the hearing loss results. As a matter of fact, the models tested were not acceptable by the structural equation model criteria used. What is also true of the above two data sets is that the hearing losses were only of the mild to moderate kinds (assessed by the pure tone audiogram in Rönnberg et al., 2011, and by the digit triplets test in Rönnberg et al., 2014), suggesting that early prevention with hearing aids should be employed (Arlinger, 2003), although the data for treatment by hearing aids is relatively meager when it comes to dementia.

At any rate, hearing loss, not visual impairment, is a very sensitive predictor variable of especially ELTM impairment, hearing loss being the largest modifiable factor of the development of dementia (Livingston et al., 2017). However, that is the overall picture and the more specific underlying mechanism as to why hearing loss is a risk factor for dementia is still argued to be unclear (Wayne and Johnsrude, 2015; Hewitt, 2017). Other independent analyses from the UK Biobank resource suggest that subclinical small variations in hearing acuity may still be associated with loss of gray matter volumes in the brain, especially in areas related to cognition and hearing (Rudner et al., 2019; however, see further about brain atrophy and cognitive reserve Uchida et al., 2021).

## Generalizations

The previously mentioned study by Marsja et al. (2022) suggests impressively similar (if not identical) cognitive predictions from one hearing-impaired group compared to a normal-hearing group on a matrix sentence latent construct. This finding suggests a powerful generalization of the case that Cognitive Hearing Science—and the ELU model—applies to anyone, regardless of hearing status.

Moreover, recent studies of different speech distortions (Kennedy-Higgins et al., 2020) show that WM and vocabulary (i.e., SLTM) come out as the main predictors, irrespective of the type of distortion (time-compressed and noise-vocoded signals, and speech in noise). This informs us that the cognitive machinery underlying speech perception and speech understanding is rather invariant in its reliance on certain cognitive building blocks irrespective of how underspecified or distorted target stimuli are. As already argued, it presupposes that rapid abstraction into formats suitable for WM is a prerequisite for the system to work.

An interesting extension of the generalization aspect is the study by Blomberg et al. (2019) of adults with Attention Deficit Hyperactivity Disorder (ADHD). She also used different kinds of speech distortions (normal vs. noise-vocoded), orthogonally combined with type of background noise (clear speech, white noise, and speech babble). Materials were taken from the Swedish HINT sentence corpus, which consists of everyday sentences (Hällgren et al., 2006). Results showed that compared to an age-matched control group there was no interaction between group and type of masker or stimulus distortion (but main effects were observed), generalizing the Kennedy-Higgins et al. (2020) findings to another group of participants, with similar kinds of distortion manipulations. This pattern may depend on the possibility that the cognitive analysis and representations are multimodal and information-based rather than modality-specific. Importantly, different assessments of WM were used to construct a cognitive factor that heavily influenced performance across the distortion/noise conditions, supporting the ELU model.

As long as information collated or bound by RAMBPHO is incomplete in some of the many ways that will cause mismatch, dependence of WM tests indicate that a certain level of generalization is possible to make. But, we would not argue that RAMBPHO processing of e.g., vocoded speech is exactly the same as e.g., RAMBPHO processing of rapid wide dynamic range compression of speech. The general point is that at a cognitive postdiction level you must (for different reasons) infer, manipulate, and “mentally fill in” some pieces of information that demand WM processing, as well as retrieval of LTM information, to reconstruct poorly specified stimulus materials. Any other model that emphasizes the cognitive work needed in degraded, distorted, or perceptually demanding conditions would also be supported by such findings across groups and stimulus conditions (e.g., the FUEL framework, Pichora-Fuller et al., 2016).

Finally, another experiment seems to indicate that load on WM is reflected in larger pupil dilation responses (assumed to reflect cognitive load) than the physical characteristics (SNR) of the task (Zekveld et al., 2018), implying that high level cognitive processing in WM is accomplished, but pushes the system to its limits for participants with low WMC. This study was followed up by a study on an auditory Stroop task (measuring executive control), where pupil size was higher in conflict conditions (e.g., saying “left” in the right ear). This connects well with our early observations that not only did WMC play an important role in speech understanding, but also executive functions or cognitive control seemed important (Badre, 2021). In ELU terms, the postdictive phase of inferring what was uttered, may use both the processing capacities of WM but also of related or overlapping executive and cognitive control functions.

## Testing the boundaries of the ELU model

Neuroimaging studies show that sensory deprivation, e.g., deafness, during development cause reorganization of superior temporal regions (auditory cortex; Bavelier and Neville, 2002; Merabet and Pascual-Leone, 2010; Andin and Holmer, 2022). Theories behind such cross-modal reorganization differs, with some suggesting pure neural processes and some suggesting behaviorally driven processes. In the case of early deafness, the latter has gained most attention, with two main lines of explanations (see extensive review in Cardin et al., 2020). The first explanation proposes functional preservation, where the type of processing in a sensory deprived region, i.e., auditory cortex, is preserved but applied to a different modality (e.g., visual instead of auditory). This notion finds support in results suggesting that superior temporal regions, which respond to speech in hearing individuals, are activated in response to sign language in deaf but not hearing signers (MacSweeney et al., 2001; Cardin et al., 2013). Such reorganization supports an extension of the ELU model to the manual-visual language modality.

The second proposal is that reorganization reflects a functional shift. This idea is supported by studies reporting activation in superior temporal regions during cognitive tasks (Twomey et al., 2017), and suggests modality-dependent differences in cognitive processes. This perspective speaks against one of the original claims of the ELU model, i.e., that there is a modality-independent “language processor” in the brain. This is of course given that superior temporal regions are exclusively engaged in language processing. However, the empirical evidence to date lends support for both explanations and it has also been suggested that they can coexist (Cardin et al., 2020).

In WM studies using sign-language material (e.g., Rönnberg et al., 2004; Bola et al., 2017; Cardin et al., 2018; Andin et al., 2021), the superior temporal regions (auditory cortex) and occipito-parietal regions (in speech-sign bilinguals, Rönnberg et al., 2004) are activated to a greater extent for deaf compared to hearing individuals. However, in a recent neuroimaging study from our lab, we found that the activation of auditory cortex did not increase with increasing WM load in a sign language-based task, suggesting a general sensory-perceptual processing role in response to visual linguistic material (Andin et al., 2021) in line with functional preservation. Further, we found support of a modality-specific pattern in relation to the degradation of the sign-language signal. In previous studies on auditory signal degradation in individuals with normal-hearing and impaired hearing, similar changes in neural activation have been identified for both increased WM load (amount of information needed to be kept in memory) and acoustic degradation. These findings have been taken as evidence for resource models of WM in general and the ELU model for language processing in particular (Obleser et al., 2012; Petersen et al., 2015; Peelle, 2018; Rönnberg et al., 2019). Although visual degradation of the language signal resulted in similar effects at the behavioral level, the neural overlap was absent for sign language in deaf early signers (Andin et al., 2021). Hence, while increasing WM load was reflected in increased engagement of the frontoparietal working memory network, as predicted, the degradation of the visual signal instead caused activation of bilateral inferior occipital and temporal cortices. The lack of neural overlap, might challenge the validity of the ELU model, potentially reflecting modality-specificity. However, it should be noted that the same effect was found for hearing non-signers. Hence, the effect might be related to presentation modality rather than the language modality. Further studies investigating the auditory and visual domain within the same paradigm are needed to further evaluate the modality-generality of the ELU model.

## Conclusion

1. Cognitive and communicative data patterns preceding the formulation of the ELU model (Rönnberg, 2003) were described. Individual cognitive ability was (and is) important for communicative competence.
2. Rapid multimodal and multilevel abstraction by means of RAMBPHO is supported by recent and previous experiments. WM stores these types of information in an on-line “moving window.”
3. Parallel levels of mismatch negativity make the system extremely effective and rapid in deconstruction and reconstruction, prediction and postdiction.
4. A use-disuse principle was introduced and combined with a multimodal memory systems account to suggest why hearing loss strikes at ELTM, SLTM, and WM in that order of decreasing negative impact.
5. Recent preliminary modelling gives strong and more nuanced support of a mediation model of the original ELU parameters, which takes into account that processing speed is important for WM operations only in adverse SPIN conditions Phonology (i.e., RAMBPHO) is a basic predictor variable of SPIN performance under all circumstances.
6. New models, such as the D-ELU were discussed. SLTM adaptations show acclimatization to certain non-habitual signal processing strategies, as well as to “perceptual doping.”
7. Language proficiency and bilingualism are further factors discussed in the D-ELU context.
8. Generalization studies have shown that hearing-impaired and normal hearing persons equally on a cognition factor as predictor of SPIN performance. Moreover, the reliance on WM across different signal distortion conditions is equal when comparing persons with ADHD and normal hearing persons.
9. Boundary conditions are discussed in a sign language context in terms of preserved brain functions which are applied to another language modality; or in terms of a functional shift, where deaf participants’ sign language use is assumed to change brain organization.

## Author contributions

JR conceived and wrote a primary draft of ELU-related research and model development. JA was especially responsible for the sign language research. CS was responsible for the ERP and MEG studies. EH was responsible for overall coherence and the D-ELU model. All authors contributed to the article and approved the submitted version.
